# Fatty Acid Profile and Enterolactone Content of Early and Commercial Milk of Dairy Cows Supplemented with Flaked Flaxseed during the Dry Period

**DOI:** 10.3390/ani10122394

**Published:** 2020-12-15

**Authors:** Lucia Bailoni, Laura Da Dalt, Gianfranco Gabai, Elisa Giaretta, Nadia Guzzo, Roberto Mantovani

**Affiliations:** 1 Department of Comparative Biomedicine and Food Science (BCA), University of Padova, Viale dell’Università 16, 35020 Legnaro, Italy; lucia.bailoni@unipd.it (L.B.); laura.dadalt@unipd.it (L.D.D.); gianfranco.gabai@unipd.it (G.G.); nadia.guzzo@unipd.it (N.G.); 2 Department of Agronomy, Food, Natural resources, Animals and Environment (DAFNAE), University of Padova, Viale dell’Università 16, 35020 Legnaro, Italy; roberto.mantovani@unipd.it

**Keywords:** flaxseed, dairy cows, dry period, enterolactone, milk fatty acids

## Abstract

**Simple Summary:**

Several studies have been published on n-3 fatty acids enrichment of food of animal origin by the use of specific supplements in animal feeding. Flaxseed (*Linum usitatissimum*) is known as an excellent source of unsaturated fatty acids, particularly of alpha-linolenic acid. Moreover, most of the plant lignans contained in flaxseeds are converted by the animal in mammalian lignans, such as enterodiol and enterolactone. Thus, milk with elevated enterolactone may be an efficient strategy to optimize the effects of lignans on human health. This study aimed to investigate the effect of flacked flaxseed supplementation on dairy cow during the pre-partum period. The increase of n-3 on milk obtained from cow fed the flaxseed diet was detected only in the first days of lactation. In addition, an increase of enterolactone on milk from flaxseed fed cows was observed only at 15th day after calving; a higher amount of enetrolactone was detected in plasma and milk from the 15th day after calving, independently from the treatment group. This study suggests that the duration of n-3 carry-over into the milk is dependent on the n-3 feeding source concentration. Finally, more studies are needed to investigate enterolactone metabolism in ruminants.

**Abstract:**

Various supplementations in animal feeding have been investigate in order to enrich food of animal origin with n-3 fatty acids. Although the effects of flaxseeds inclusion in diets for lactating dairy have already been assessed, few studies have focused on this n-3 source supplementation during the transition period. The aim of this work was to evaluate the effects of flacked flaxseed (200 g/head/day; 2.13% DM) dietary treatment during the dry period on milk yield and quality in the 30 days after calving. In addition, the enterolactone content in plasma (before and after calving) and in milk of cows fed diets supplemented or not with flaxseed was considered. The study demonstrated that the carry-over effect on the milk profile of C18:2, C18:3 n-3, and C20:5 n-3 was significantly higher in flaxseed diet than in the control one at 4th day of lactation. A significant increase of enterolactone on milk from flaxseed fed cows was observed only at 15 sampling day. The quick modification in fatty acid (FA) profile of the milk in the first few days of lactation suggests that the carry over effect from pre-calving flaxseed feeding at this concentration was very short lasting.

## 1. Introduction

In human nutrition, the beneficial effects of n-3 fatty acids (FA; i.e., a decreased risk of cardiovascular diseases, hypertension, and arthritis) are well known from years and dietary inclusion of these nutrients is recommended [1,2]. As a consequence, a large number of studies have been published on n-3 FA enrichment of food of animal origin (milk, meat, eggs, and fish) by the use of specific supplements in animal feeding [3,4,5,6]. Flaxseed (*Linum usitatissimum*) is known as an excellent source of unsaturated fatty acids (FA), particularly of alpha-linolenic acid (ALA, C18:3n-3), a member of n-3 FA [2]. A large number of studies have reported the results of flaxseeds inclusion in diets for lactating dairy cows finalized to enrich milk [7,8,9] and derivate [10] in n-3 FA. In addition, several studies have shown that the incorporation of dietary n-3 into ovarian structures and bull sperm influence the reproductive features in terms of follicular and embryo development, and bull semen quality [11]. It was also demonstrated that the supplementation of n-3 FA during the early postpartum period modifies the endocannabinoid system in the bovine endometrium [12]. Feeding source of n-3 FA can modulate the chemical composition and the functional proprieties of the immune cells, which could affect the immune response in several ways [11].

To our knowledge, most of the studies have analyzed how flaxseed supplementation during only the dry period could impact on subsequent reproductive performance, bloody metabolites, and milk yield and quality during the subsequent lactation [13,14,15]. However, few studies have focused on the effects of flaxseed supplementation during the transition period on milk composition and FA metabolism [16,17,18].

Several studies demonstrated the beneficial effects of lignans on human health [7]. Plant lignans contained in flaxseed (about 370 mg/100 dry matter; DM) have a lower bioavailability in the human intestine compared with the mammalian lignans synthetized in the rumen [19]. The secoisolariciresinol diglucoside (SDG), which represents over 95% of the total lignans in flaxseed, could be converted by the rumen microorganisms [20] in enterodiol and enterolactone (EL). Recent studies demonstrated that flaxseed dietary treatment could increment the amount of milk EL [7], which in this way, could be considered as a valid source of mammalian lignans.

Following this rationale, the objectives of the present experiment were i) to evaluate the effects on milk yield and quality in early lactation and commercial milk due to the supplementation of dairy cows with flacked flaxseed during the dry period and ii) to evaluate the EL content in plasma and milk of cows fed diets supplemented or not with flaxseed during the dry period.

## 2. Materials and Methods

### 2.1. Ethic Statement

All experimental procedures were carried out according to Italian law on animal care (Legislative Decree No. 26 of 14 March 2014) and approved by the ethical committee at the University of Padova (approval number 70/2019).

### 2.2. Animals and Feeeding

The study was conducted in a commercial dairy farm located in Crespano del Grappa in the province of Treviso, northeast of Italy. Italian Friesian cows with a close date of drying off were divided in two homogeneous groups for parity (2.25 ± 1.72), days open (117 ± 66), and milk production level in the current lactation (9.858 ± 1.598 kg of mature equivalent milk). Couples of cows at the beginning of the dry off period were randomly assigned to receive a control (CTR) or an experimental flaxseed (FLAX) supplement diet by assigning them to different pens to allow the proper feeding supplementation. At the end of the trial, cows used in the experiment were 38 in the CTR and 35 in the FLAX group. During the dry period, the cows of the FLAX group were fed a diet supplemented with 200 g/head/day (i.e., 2.13% of DM) of flacked flaxseed (Cortal Extrasoy, Cittadella, Padova, Italy) and the CTR group received a mix of soybean and corn meals in order to obtain isoenergetic and isoproteic rations. The steaming-up treatment was managed in both groups following the customary farm procedures. After calving, lactating cows of both groups were fed ad libitum the same total mixed ration (TMR). Diets of dry and lactating cows were formulated in order to satisfy the nutritional requirements reported by the National Research Council—NRC [21]. During the trial, cows had free access to water and were fed once daily.

### 2.3. Data and Sample Collection

Samples of the single ingredients belonging to each diet (except the flaked flaxseed) were collected every 2 months, immediately transferred to the laboratory and, if fresh, subjected to pre-drying, before the analyses (overall 4 samplings were carried out during the experiment). The chemical composition of CTR and FLAX diets during the dry period and lactation diet after calving was calculated on the basis of the results of the chemical analyses of feed and the changes of the diet formulation over time. Diets were prepared and distributed as total mixed rations (TMR) using a mixed wagon (Table 1).

Flacked flaxseed was collected at each change of the batch (for a total of 7 samples) to be analyzed for chemical composition and FA profile (Table 2).

Daily milk yield was recorded in the first 30 days after calving using an automatic milking system for a herringbone parlor coupled with an automatic recording software (ALPROTM by DeLaval^©^, Tumba, Sweden). Individual milk samples (500 mL/each) from the morning milking were collected from each cow at about 4, 15, and 30 days after calving to be analyzed for composition, FA profile, and the enterolactone content.

Individual milk samples (500 mL/each) from the morning milking were collected from each cow at 4.1 ± 1.5, 15.5 ± 1.6, and 30.9 ± 1.6 days after calving. Blood samples were collected from each animal at about 54.9 ± 6.7, 32.2 ± 10.6, 9.2 ± 2.5, and 3.1 ± 1.5 days before the expected calving and at 4.1 ± 1.5, 15.5 ± 1.6, and 30.9 ± 1.6 days after calving. The samples were obtained from the jugular vein in vacuum tubes containing K3-EDTA anticoagulant and without anticoagulant. Plasma and serum were obtained by centrifugation (1.500× *g* for 15 min at 4 °C) and an aliquot of each sample was frozen and stored at −20 °C for enterolactone content analysis.

### 2.4. Sample Analysis

Samples of feeds were analyzed for dry matter (DM: # 934.01; [22]), N (# 976.05; [22]), EE (# 920.29; [22]), and ash (# 942.05; [22]). Neutral detergent fiber (NDF), inclusive of residual ash, was determined with α-amylase using the Ankom220 Fiber Analyzer (Ankom Technology, Macedon, NY, USA). Acid detergent fiber (ADF), inclusive of residual ash, was determined sequentially after NDF determination [23]. Starch content was determined after hydrolysis to glucose [22] by liquid chromatography [24]. The flacked flaxseed fat was extracted by accelerated solvent extraction (ASE 200, Dionex Corp., Sunnyvale, CA, USA) using petroleum ether. The fatty acid profile of the flaxseed samples was analyzed by GC with flame-ionization detector (7890A GC system, Agilent Technologies, Milan, Italy) using two columns in series and equipped with a modulator (Agilent G3486 A CFT), an automatic sampler (Agilent 7693), and a specific machine software (Agilent Chem Station). This instrument was chosen because the double column allows separating and identifying each FA on a 2-dimensional basis [25]. The first column was a 75 m × 180 μm (internal diameter) × 0.14 μm film thickness column (23348U, Supelco, Bellefonte, PA, USA), the second was a 3.8 m × 250 μm (internal diameter) × 0.25 μm film thickness column (J&W 19091-L431, Agilent Technologies, Santa Clara, USA). Both columns used H2 as carrier gas at a flow rate of 0.22 mL/min.

The early and commercial milk samples were analyzed for fat, protein, casein, and lactose contents using the FIL-IDF procedure [26] with MilkoScan™ FT1 apparatus (Foss Electric, DK-3400, Hillerød, Denmark). Milk urea nitrogen was measured automatically by the conduct metric-enzymatic method (CL 10 micro analyzer, Eurochem, Roma, Italy). Somatic cell count was carried out through a FossomaticTM 5000 (Foss Electric, DK-3400, Hillerød, Denmark) according to the standard FIL-IDF148a [27], and transformed in logarithmic terms using the following equation: SCS = 3 + log_2_ (SCC/100.000).

Milk fat was extracted by accelerated solvent extraction (ASE 200, Dionex Corp., Sunnyvale, CA, USA) using petroleum ether:isopropanol (3:2 vol/vol), and the extracted fat was analyzed for FA composition using a two-dimensional gas-chromatography instrument (Agilent 7890A, Agilent Technologies, Milan, Italy) as described above.

Enterolactone concentration in plasma and milk samples was measured following hydrolysis and solvent extraction. Briefly, 200 μL of plasma and 500 μL milk were hydrolyzed respectively in 200 or 500 μL of acetate buffer (0.1 M. pH 5.0) containing 0.2 U/mL of β-glucuronidase and 2 U/mL of arylsulfatase (Sigma-Aldrich, S. Louis, MO, USA) as described by Stumpf et al., 2000 [28]. Samples were incubated overnight for plasma and 1.5 h for milk at 37 °C. Only the milk samples were washed with 3 mL of hexane to remove lipids as described by [29]. Then, enterolactone was extracted twice with 2 mL of diethyl ether in both plasma and milk. The dry extracts were dissolved in 200 and 500 μL of assay buffer respectively (TR-FIA Enterolactone kit, Labmaster, Finland) and assayed in duplicate by a commercial DELFIA method (TR-FIA Enterolactone kit, Labmaster, Finland). For the final assay, extracts corresponding to 20 μL of plasma/milk were used. Fluorescence was read in a multilabel reader (Victor X4 2030, Perkin-Elmer Instruments, Norwalk, CT, USA).

### 2.5. Statistical Analysis

Daily milk production, milk quality data, and fatty acid profile of milk were analyzed using a hierarchical linear model for repeated measures implemented through the PROC MIXED of SAS [30], according to the following linear model:

y_ijklm_ = μ + D_i_ + P_j_ + DP_ij_ + C_k:ij_ + S_l_ + DS_il_ + PS_jl_ + e_ijklm_, (1)

where µ is the overall mean; D_i_ is the fixed effect of diet (i = 2 levels: CTR and FLAX); P_j_ is the fixed effect of parity (j = 3 levels: 2nd lactation, 3rd lactation, and more than 3rd lactations); DP_ij_ is the fixed effect of the interaction between diet i and parity j; C_k:ij_ is the random effect of the cow within DP ~N(0, σ^2^_c_) used as error term for D, P, and DP; S_l_ is the fixed effect of sampling days (l = 1–30 levels for milk yield; l = 3 levels i.e., 4, 15, and 30 days after calving for milk quality and fatty acids); DS_il_ is the fixed effect of interaction between diet i and sampling days l; PS_jl_ is the fixed effect of interaction between parity j and sampling days l; e_ijklm_ is the random residual term ~N(0, σ^2^_e_).

Data on enterolactone concentration was analyzed using the same hierarchical linear model as above, but considering also individual SCS as linear covariate.

In all statistical analyses, *p* < 0.05 indicates significance and *p* < 0.10 and ≥ 0.05 indicate a tendency toward significance

When multiple comparison tests were carried out for selected variables, the Bonferroni correction method was applied [30].

## 3. Results

The results are reported considering the milk yield during the first month of lactation, the quality and the fatty acids profile of milk, and the content of enterolactone in plasma and milk.

### 3.1. Milk Yield

**Figure 1:**
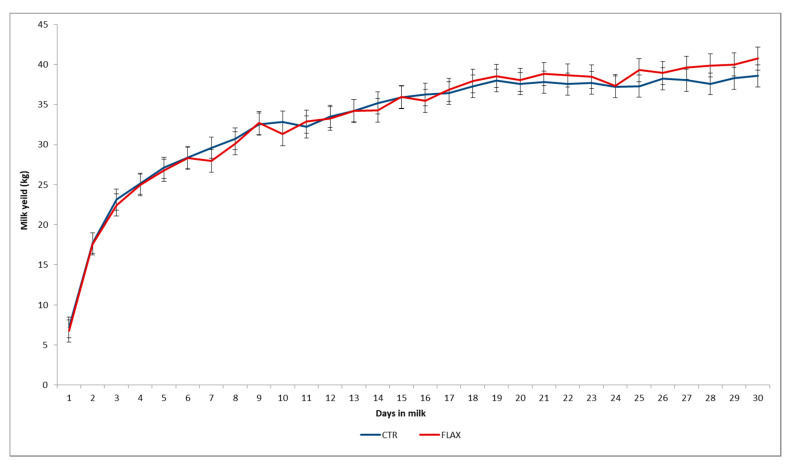
Least square means for control (CTR; solid line) and flaxseed supplemented (FLAX; dotted line) diets on the early and commercial milk production of Holstein cows during the first 30 d of the subsequent lactation.

The production of early and commercial milk during the first month of lactation was similar for the cows fed with or without flaxseed during the dry period (Figure 1). The overall milk yield of cows of the control group (CTR) and the cows receiving flaxseed (FLAX) was respectively 989 ± 41 kg and 998 ± 43 kg (*p* ≥ 0.05).

There were no differences in overall milk yield produced during the first 30 days after calving between the cows of 2nd, 3rd, or more than 3rd lactation (*p* ≥ 0.05). The effect of interaction between diet and parity was not significant.

### 3.2. Quality of Early and Commercial Milk

The effect of the diet, sampling day, and parity on the quality of early and commercial milk is shown in Table 3.

There were no significant differences in all parameters of milk quality between the CTR and FLAX diet (Table 3).

On the contrary, the sampling day affected significantly (*p* < 0.001) all quality parameters of early and commercial milk (Table 3). The fat, protein, and casein content decreased passing from 4 (early milk) to 15 and 30 days in milk (*p* < 0.001). The lactose content increased from 4 to 15 and 30 days of lactation (*p* < 0.05). The somatic cell score (SCS) of early and commercial milk produced after 4 DIM was higher than those of milk sampled subsequently (*p* < 0.001). Lastly, the milk urea nitrogen (MUN) increased passing from 4 to 15 and 30 day of sampling (*p* < 0.001).

The differences in early and commercial milk quality parameters were not significant (*p* ≥ 0.05) among early and commercial milk of cows different in parity (Table 3).

All effects of the first order interactions were not significant.

### 3.3. Fatty Acid Profile of Early and Commercial Milk

The Effect of the Diet, Sampling Day and Parity on the Fatty Acid Profile of Early and Commercial Milk Is Shown in Table 4

No differences (*p* ≥ 0.05) were observed in saturated fatty acids (SFA) and in monounsaturated fatty acids (MUFA) contents between CTR and FLAX experimental groups, except for C17:1 n-10. Considering the polyunsaturated fatty acids (PUFA), the levels of C18:2 (linoleic acid), C18:3 n3 (alpha linolenic acid, ALA), and C20:5 n3 (eicosapentaenoic acid, EPA) were higher (*p* < 0.05) in early and commercial milk obtained by cows receiving flaxseed during dry period (FLAX) than that produced by cows of the control (CTR) group. Otherwise, C20:3 n6 was significantly lower in FLAX than in CTR. Consequently, the total amount of n3 fatty acid was higher in FLAX than in CTR group (*p* = 0.02) and the ratio between n6 and n3 fatty acids resulted more favorable (*p* < 0.01) in the early and commercial milk obtained from cows receiving flax during the dry period.

The sampling day affected significantly (*p* < 0.001) most of the fatty acids reported in Table 4. The content of the total saturated FA was lower at 30 days in milk than at the beginning of lactation (day 4), while the pattern of monounsaturated FA was opposite (*p* < 0.001). The content of polyunsaturated FA was lower in early milk (4 day sampling) compared with the subsequent samplings (*p* < 0.001). ALA and total n3 fatty acid content resulted higher in the intermediate sampling (15 d) than in the first and last samplings (*p* < 0.001). The n6:n3 ratio resulted more favorable in the intermediate sampling (15 d) than at 4 or 30 d of sampling (*p* < 0.001).

The effect of parity was not significant (*p* ≥ 0.05) for the most of fatty acids showed in Table 4, excluding C18:0 (stearic acid), C12:1, C18:1 trans n-7, and C18:1 cis n-2 (*p* < 0.05). The conjugated linoleic acid isomer (CLA) named C18:2 cis-9, trans-11 resulted higher (*p* < 0.05) in early milk (first sampling) than in the two other samplings (15 and 30 d).

**Figure 2:**
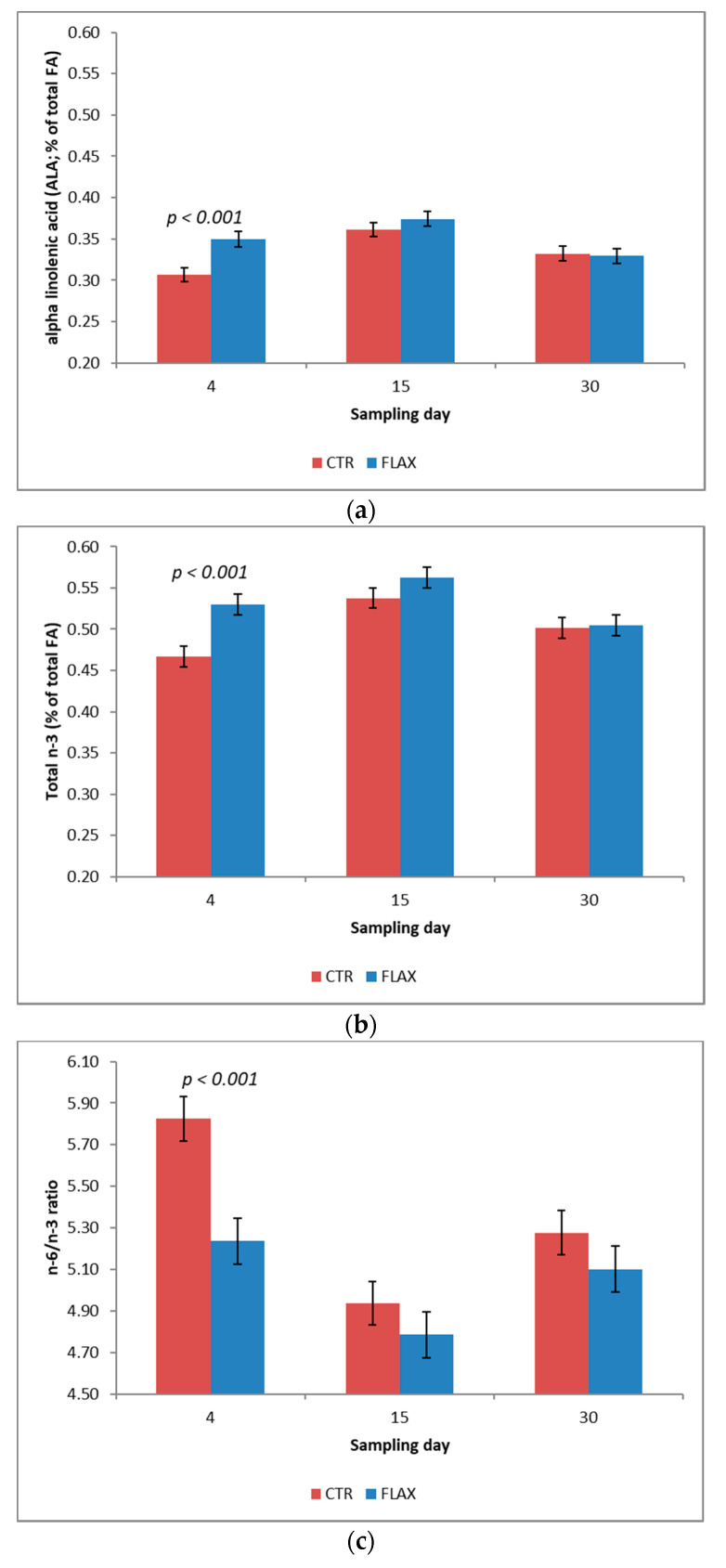
Least square means for control (CTR; dark red bars) and flaxseed supplemented diets (FLAX; blue bars): (**a**) alpha linolenic acid (ALA); (**b**) total n-3 fatty acids contents (expressed as % on the total fatty acids); and (**c**) n-6: n-3 ratio in the early and commercial milk. Differences between treatments within sampling day have been reported when significant (*p* < 0.05 or less).

No interactions between dietary treatment and sampling period were observed in the fatty acids analyzed, except for ALA, the sum of n3 fatty acids, and the n6:n3 ratio, which were higher (*p* < 0.001) in the early milk (4 day of sampling) of the FLAX group than of the CTR one (Figure 2A–C).

The fatty acids profile was unaffected by the interaction between diet and parity. The effect of the interaction between sampling day and parity was significant for some polyunsaturated fatty acids (data not shown).

Figure 2 reports the interaction between diets and sampling day effects, highlighting the significant differences between treatments within sampling days.

### 3.4. Enterolactone in Plasma and Early and Commercial Milk

**Figure 3:**
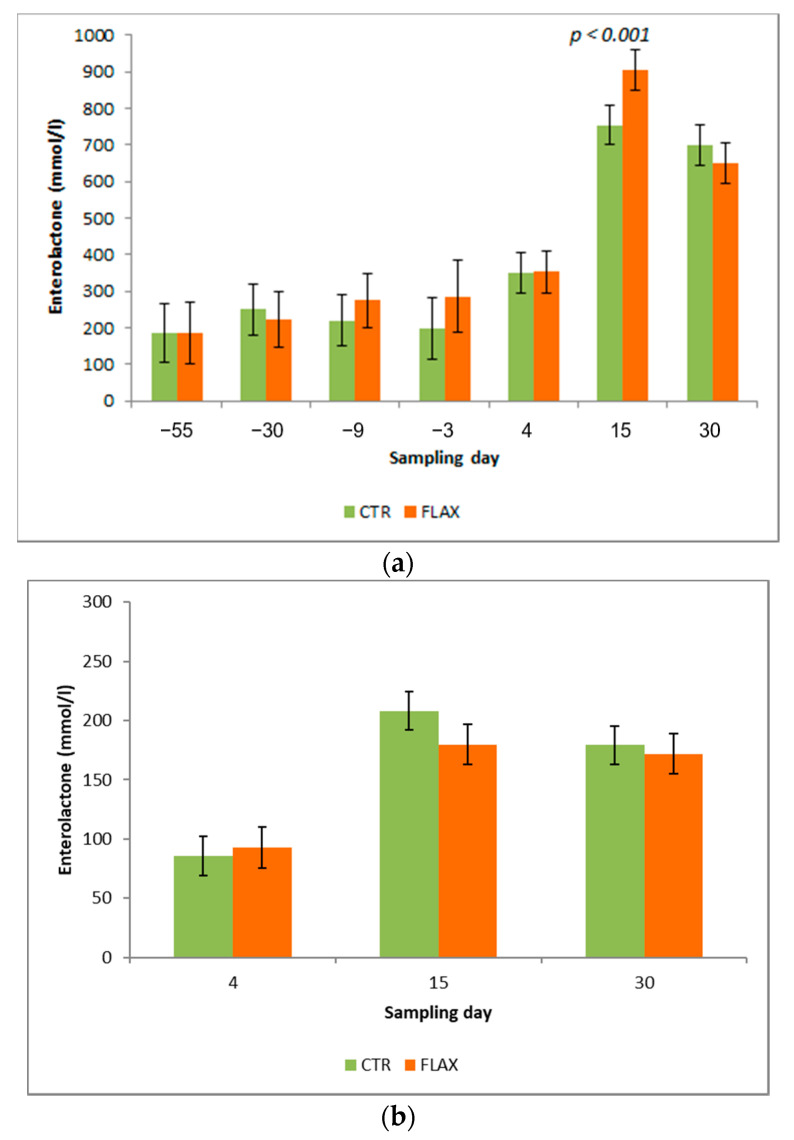
Concentration (nmol/L) of enterolactone in Holstein cows fed either with flacked flaxseed or a control diet during the dry period: (**a**) concentration in plasma (before and 30 d after calving); (**b**) concentration in milk during 30 d after calving. Differences between treatments within sampling day have been reported when significant (*p* < 0.05).

The enterolactone concentration (EL, nmol/L) was detected in plasma and in early and commercial milk (Figure 3). No significant differences were observed between diets for the concentration of enterolactone in plasma, although the effect of sampling day was significant (*p* < 0.001; data not shown). The EL concentration in plasma samples collected before calving (4 sampling days) was lower than those of samples collected during the first month of lactation (3 sampling days; *p* < 0.001; data not presented). The parity of cows had no effect of EL. All interactions of first order were not significant. Therefore, the supplementation of FLAX did not affect the EL concentration during the dry period and the subsequent lactation. The only exception was for the interaction between diet and sampling day. Indeed, EL concentration in plasma was greater in FLAX fed than in CTR cows at d 15 after calving (*p* < 0.001; Figure 3a).

There were no significant effects (*p* ≥ 0.05) on EL in milk for the two experimental groups (CTR and FLAX). On the contrary, the EL concentration increased significantly (*p* < 0.001) in early and commercial milk from 4 to 15 days of lactation, remaining high at 30 day after calving. No differences were observed among the cows of different parity. All interactions of first order were not significant.

## 4. Discussion

Supplementation with n-3 FA of cattle feeding is an effective approach for improving the nutritional value of animal products such as meat and milk [3,15,31,32]. Many studies [9,10,17,32] demonstrated that flaxseed supplementation in cows during lactation or transition periods significantly increase the amount of n-3 FA in milk, which is otherwise relatively poor in these FA [33]. Nevertheless, very few studies have been conducted on the effects of pre-partum n-3 supplementation on milk composition [13,15].

During early lactation, dairy cows experience a negative energy balance associated with mobilization of body reserves. In this period, dairy cows are in negative energy balance, causing extensive body-fat mobilization and their incorporation into milk fat. Thus, milk FA synthesized de novo by the mammary gland is lower, compared to mid or late lactation [15,34] and depends on FA composition of adipose tissue [35]. n-3 FA cannot be synthesized de novo by mammals and is referred to as essential fatty acids, which must be acquired with the diet [36]. Our study demonstrated that the carry-over effect on the milk profile of C18:2, C18:3 n-3, and C20:5 n-3 (EPA) was significantly higher in FLAX diet than in the CTR one on the first sampling at 4th day of lactation. Present results are in agreement with early studies reporting that extruded or crushed linseeds (2.5% or 3.3% DM) supplemented on transition period increased 18:3 n-3, and total n-3 concentrations in early milk, but did not exert any effect after 7 weeks of lactation [32,37]. In contrast with our results, a study [15] demonstrated that the carry-over effects persisted for 7 weeks of lactation (10 weeks after the withdrawal of extruded flaxseed supplementation, 2.9% DM). However, the greater amount of n-3 was observed during the first week with respect to the other lactation phases. In contrast, another study has shown that restricted diet with canola, linola, or flax rolled seeds supplementation (8% DM) in the 4 last weeks of gestation had no effects on fatty acids milk compositions and metabolic peri-partum responses [13].

These different results could be related to the different flaxseed treatments and concentration used in these studies [38]. Lipids in extruded flaxseed are considered to be at least partially rumen-protected and thus can overtake the microbial bio-hydrogenation [39,40], while unprocessed oilseeds have a less influence on supplying n-3 on meat or milk [35,41]. Although various studies have been performed on the effect of different processing treatment (grounding vs. term-flaking) on cows performance and feed utilization [42,43], the lipid and FA metabolism of enriched n-3 sources subjected to different physical treatments has not yet been deepened. Regarding the amount of oilseed supplementation, in our FLAX diet, flaked flaxseeds was added at 2.13% of DM, thus in a lower concentration than the previous studies. This difference and the length of supplementation, which only covered the dry period, may have determined the differences due to flaxseed supplementation compared to the other studies.

The quick variation in FA profile of the milk in the first few days of lactation observed in our study suggests that the carry-over effect from pre-calving flaxseed feeding at this concentration was very short lasting. In addition, an interesting result of our study is the significant increase of EPA, resulting from ALA elongation, on early milk of cows fed the FLAX diet [44]. Previous findings have shown that conversion of linoleic acid (LA) and alpha-linolenic acid (ALA) to their higher chain homologues (DHA and EPA) in humans depends on the ratio of ingested n-6 and n-3 fatty acids [44]. Twenty-carbon PUFAs are precursors of eicosanoids that regulate the inflammatory and immune responses through pro- and anti-inflammatory activities [36]. In this sense, n-3 supplementation on pre-partum diet could be considered as a valid approach to improve the nutritional value of early milk and consequently calf health. Indeed, as recently demonstrated by [45], pre-partum fat feeding modified the FA profile of early milk to a greater degree than plasma of newborns, suggesting that the metabolism and transfer of essential FA from the mammary gland to the early milk has a less tight regulation than those from the placenta to the fetus. Thus, providing early milk with selected FA through pre-calving dietary supplementation could be an efficient strategy to improve calf health status immediately after birth.

Our results demonstrated that, independently from treatment group, plasma and milk EL significantly increase 15 days after calving and it remains high for the consecutive 15 days postpartum. According to [19] study, plant and mammalian lignans have different bioavailability in humans: while the first must be converted to EL by the colon bacteria, the others can be passively absorbed along the human intestine. Therefore, milk with an elevated amount of EL may be an efficient strategy to optimize the effects of lignans on human health [46]. According to our results, a significant increase of EL on milk from FLAX-fed cows was observed only at 15 sampling day. Four dose-response studies using flax hulls (FH), flax meal (FM), and whole flaxseed (WF) have been conducted to date [7,46,47,48]. In these studies, higher concentration of milk EL was observed with FH or FM diet supplementation compared with WF feeding. In addition, a significant increase of milk EL concentration when cows were fed the greatest amount of flaxseed supplementation (15% FM or 20% FH on DM basis) [8]. However, these higher amounts of flaxseed products compared with that used in our study may have multiple adverse effects associated with the excess intake of crude protein or crude fat. Indeed, the excess of N due to environmental and milk production concerns associated with excess intake of crude protein or crude fat, depending on the flax source used [8]. A recent study demonstrated that calves fed EL-enriched milk (481 nmol/L) showed a faster and higher EL increase on plasma, compared with those fed milk enriched with a lower amount of EL (123 nmol/L). Further research is needed to better understand the physiological EL concentration on milk and plasma and its metabolism before and after calving, in order to find the balanced intake of oilseed supplements on dairy cow dietary treatment.

## 5. Conclusions

Fatty acid profiles of early milk samples were influenced by flaked flaxseed supplementation provided to dry cows. However, the effect obtained from the concentration used in this study (2.13% DM) was very short lasting. These results suggest that different concentrations and the various treatments, to which flaxseed are subjected, significantly affect the n-3 carry over into the milk. Differences of EL plasma concentration before and after calving should be better investigated, in order to understand EL metabolism in ruminants and how it could be efficiently manipulated by dietary treatments.

## Figures and Tables

**Table 1:** Chemical composition (mean ad standard deviation, SD) of control (CTR) and flaxseed supplemented (FLAX) diets used during the trial expressed as % on DM basis.

Chemical Composition	Dry Period				Lactation Period	
	CTR ^1^		FLAX ^2^			
	Mean	SD	Mean	SD	Mean	SD
DM, %	49.0	8.47	49.4	8.49	54.3	4.70
Crude protein	12.2	0.76	12.4	0.73	16.7	0.30
Ether extract	2.64	0.64	3.10	0.68	4.80	0.60
Ash	6.82	1.08	6.75	1.06	5.86	0.71
NDF	49.1	6.56	48.5	6.47	34.0	4.10
ADF	28.1	4.30	27.7	4.22	18.9	2.91
Starch	12.2	2.85	12.0	2.79	24.2	2.30
MFU ^3^ no./kg DM	0.78	0.04	0.78	0.04	097	0.02

^1^ CTR: control diet, ^2^ FLAX: experimental flaxseed supplemented diet, ^3^ MFU: milk forage unit; calculated using published value of feed ingredients (INRA. 1984).

**Table 2:** Descriptive statistics (mean ad standard deviation, SD) for chemical composition (% on DM basis) and fatty acid profile (% of total FA) of the flacked flaxseed used in the study (n = 7 samples).

Chemical Composition	Mean	SD
DM, %	93.2	1.69
Crude protein	22.3	1.37
Ether extract	44.1	2.05
Ash	3.25	0.47
NDF	13.4	0.75
ADF	7.13	0.23
MFU ^1^ no./kg DM	0.98	-
Fatty acid profile		
SFA	10.9	0.75
MUFA	16.8	2.80
PUFA	67.9	3.46
n-3	53.9	3.65
C18:3 n3	53.8	3.63
n-6	15.9	0.46
n-6/n-3	0.31	0.03

^1^ MFU: milk forage unit; calculated using published value of feed ingredients (INRA. 1984).

**Table 3:** Effect of flaxseed supplementation during dry period, sampling day, and parity on the early and commercial milk quality of Holstein cows during the first 30 d of the subsequent lactation.

Item	Diet			Sampling Day				Parity				Residual
	CTR ^3^	FLAX ^4^	*p*	4	15	30	*p*	2nd	3rd	>3rd	*p*	
Milk quality												
Fat, %	4.65	4.79	0.614	6.25	4.01	3.88	<0.001	4.79	4.77	4.59	0.796	1.518
Protein, %	3.55	3.54	0.881	4.40	3.20	3.03	<0.001	3.62	3.49	3.53	0.504	0.396
Casein, %	2.75	2.75	0.968	3.35	2.50	2.40	<0.001	2.83	2.70	2.74	0.302	0.228
Lactose, %	4.72	4.69	0.431	4.47	4.79	4.85	<0.001	4.75	4.67	4.68	0.175	0.021
SCS ^1^, score	3.04	2.89	0.648	3.91	2.53	2.46	<0.001	2.56	2.97	3.37	0.078	2.352
MUN ^2^, mg/dL	18.77	18.70	0.941	15.52	19.71	20.99	<0.001	18.71	18.16	19.35	0.619	24.206

^1^ SCS = somatic cell score; ^2^ MUN = milk urea nitrogen; ^3^ CTR = control group; ^4^ FLAX = flaxseed group.

**Table 4:** Effect of flaxseed supplementation during dry period, sampling day, and parity on the fatty acid profile in early and commercial milk of Holstein cows during the first 30 d of the subsequent lactation.

Item	Diet		Sampling Day				Parity					Residual
	CTR ^4^	FLAX ^5^	*p*	4	15	30	*p*	2nd	3rd	>3rd	*p*	
SFA ^1^, % of total FA ^2^
C4:0	3.05	3.09	0.492	3.29	3.08	2.84	<0.001	3.05	3.15	3.01	0.216	0.123
C6:0	1.88	1.89	0.735	1.69	2.03	1.94	<0.001	1.93	1.85	1.87	0.256	0.056
C8:0	1.05	1.04	0.963	0.84	1.15	1.14	<0.001	1.09	1	1.05	0.122	0.028
C10:0	2.17	2.14	0.778	1.61	2.3	2.56	<0.001	2.27	2.02	2.18	0.117	0.196
C11:0	0.08	0.08	0.535	0.03	0.11	0.1	<0.001	0.09	0.07	0.08	0.137	0.001
C12:0	2.43	2.39	0.747	1.83	2.5	2.9	<0.001	2.54	2.24	2.45	0.116	0.271
C13:0	0.12	0.12	0.508	0.06	0.15	0.15	<0.001	0.13	0.11	0.12	0.133	0.002
C14:0	8.92	8.89	0.894	8.16	8.87	9.69	<0.001	9.22	8.71	8.79	0.175	2.096
C15:0	0.89	0.86	0.509	0.62	0.94	1.06	<0.001	0.91	0.85	0.85	0.251	0.037
C16:0	29.4	29.1	0.627	30.5	28.3	28.9	<0.001	29.1	29.5	29.1	0.785	6.037
C17:0	0.7	0.7	0.904	0.73	0.72	0.65	<0.001	0.7	0.7	0.7	0.889	0.008
C18:0	11.1	11.1	0.902	11.5	10.9	10.9	0.074	10.9	11.8	10.5	0.012	2.501
C19:0	0.06	0.06	0.72	0.04	0.06	0.08	<0.001	0.06	0.06	0.05	0.222	0.001
C20:0	0.15	0.15	0.466	0.11	0.17	0.18	<0.001	0.15	0.16	0.15	0.089	0.001
C22:0	0.04	0.04	0.352	0.02	0.04	0.06	<0.001	0.04	0.04	0.04	0.318	0
Others SFA ^1^	2.2	2.17	0.432	1.81	2.43	2.31	<0.001	2.23	2.14	2.2	0.227	0.05
MUFA ^3^ % of total FA ^2^												
C10:1	0.18	0.18	0.459	0.1	0.2	0.24	<0.001	0.18	0.16	0.19	0.041	0.002
C12:1	0.06	0.06	0.427	0.03	0.06	0.08	<0.001	0.06	0.05	0.06	0.026	0
C14:1	0.36	0.33	0.101	0.46	0.57	0.01	<0.001	0.35	0.32	0.36	0.228	0.016
C16:1	0.11	0.1	0.815	0.18	0.07	0.06	<0.001	0.11	0.1	0.1	0.586	0.004
C17:1n-10	0.02	0.01	0.041	0.02	0.02	0.01	<0.001	0.02	0.01	0.02	0.21	0
C17:1n-9	0.02	0.01	0.15	0.01	0.02	0.02	<0.001	0.01	0.01	0.01	0.973	0
C17:1 cis n-7	0.4	0.41	0.567	0.42	0.44	0.35	<0.001	0.4	0.38	0.42	0.3	0.005
C18:1 trans n-7	1.4	1.48	0.472	0.95	1.72	1.66	<0.001	1.62	1.42	1.28	0.028	0.544
C18:1 cis n-9	23.7	23.9	0.828	26.1	23.5	21.7	<0.001	23.1	23.7	24.57	0.231	10.01
C18:1n-6	0.32	0.32	0.995	0.22	0.36	0.39	<0.001	0.33	0.33	0.3	0.283	0.005
C18:1 cis n-2	0.06	0.06	0.964	0.04	0.07	0.08	<0.001	0.06	0.06	0.05	0.038	0
C20:1n-12	0.1	0.1	0.338	0.06	0.12	0.12	<0.001	0.1	0.1	0.1	0.695	0.001
Others MUFA ^3^	4.68	4.75	0.554	4.41	4.5	5.23	<0.001	4.67	4.64	4.83	0.471	0.214
PUFA ^6^, % of total FA												
C18:2 trans-9, cis-12 ^5^	0.031	0.035	0.424	0.03	0.04	0.03	0.346	0.04	0.03	0.03	0.034	0.001
C18:2n-6 cis	2.243	2.239	0.927	2.29	2.24	2.19	0.124	2.23	2.15	2.34	0.064	0.074
C18:2	0.022	0.017	0.04	0.03	0.02	0.01	<0.001	0.02	0.02	0.02	0.348	0
C18:2 cis-9, trans-11	0.329	0.329	0.994	0.26	0.37	0.35	<0.001	0.34	0.31	0.32	0.03	0.005
CLA ^7^												
C18:2 trans-10, cis-12	0.014	0.014	0.833	0.01	0.02	0.01	<0.001	0.01	0.01	0.01	0.862	0
CLA ^7^												
C18:3n-3	0.334	0.353	0.059	0.33	0.37	0.33	<0.001	0.34	0.33	0.35	0.317	0.002
C18:3n-6	0.05	0.046	0.219	0.04	0.05	0.05	0.001	0.05	0.05	0.05	0.213	0
C20:2	0.044	0.04	0.395	0.03	0.03	0.07	<0.001	0.04	0.04	0.04	0.831	0.001
C20:3n-6	0.12	0.108	0.035	0.1	0.12	0.12	<0.001	0.11	0.12	0.11	0.424	0.001
C20:4n-6	0.21	0.211	0.95	0.23	0.22	0.18	<0.001	0.21	0.21	0.22	0.284	0.002
C20:5n-3	0.048	0.055	0.018	0.05	0.06	0.05	0.072	0.05	0.05	0.05	0.992	0
Others PUFA ^3^	0.99	1.01	0.444	0.82	1.09	1.1	<0.001	1.01	0.97	1.02	0.426	0.022
SFA ^1^	64.2	63.9	0.695	62.8	63.8	65.5	<0.001	64.5	64.4	63.1	0.327	13.32
MUFA ^3^	31.4	31.7	0.731	33	31.7	30	<0.001	31.1	31.3	32.3	0.393	12.14
PUFA ^6^	4.42	4.47	0.609	4.22	4.61	4.51	<0.001	4.45	4.3	4.57	0.121	0.184
SFA ^1^/(MUFA ^3^ + PUFA ^6^)	1.85	1.81	0.598	1.73	1.8	1.95	<0.001	1.86	1.86	1.75	0.358	0.086
n-6	2.66	2.65	0.886	2.71	2.66	2.59	0.048	2.63	2.57	2.77	0.048	0.082
n-3	0.5	0.53	0.022	0.5	0.55	0.5	<0.001	0.52	0.51	0.52	0.757	0.004
n-6/n-3	5.35	5.04	0.006	5.53	4.86	5.19	<0.001	5.11	5.08	5.4	0.043	0.3

^1^ SFA = saturated fatty acids; ^2^ FA = FA; ^3^ MUFA = monounsaturated FA; ^4^ CTR = control group; ^5^ FLAX = flaxseed group; ^6^ PUFA = polyunsaturated FA; ^7^ CLA = conjugated linoleic acid isomers.
